# Emerging collaborations at the forefront of growth in electrochemical synthesis

**DOI:** 10.1016/j.isci.2021.102639

**Published:** 2021-06-08

**Authors:** Raffaella Buonsanti, Wilson Smith

**Affiliations:** 1 Laboratory of Nanochemistry for Energy (LNCE), Department of Chemical Sciences and Engineering, École Polytechnique Fédérale de Lausanne, Sion 1950, Switzerland; 2 National Renewable Energy Laboratory, Golden, CO 80401, USA; 3 Department of Chemical and Biological Engineering, University of Colorado Boulder, Boulder, CO 80303, USA; 4 Materials for Energy Conversion and Storage (MECS), Department of Chemical Engineering, Delft University of Technology, van der Maasweg 9, Delft, 2629 HZ, Netherlands

**Figure:**
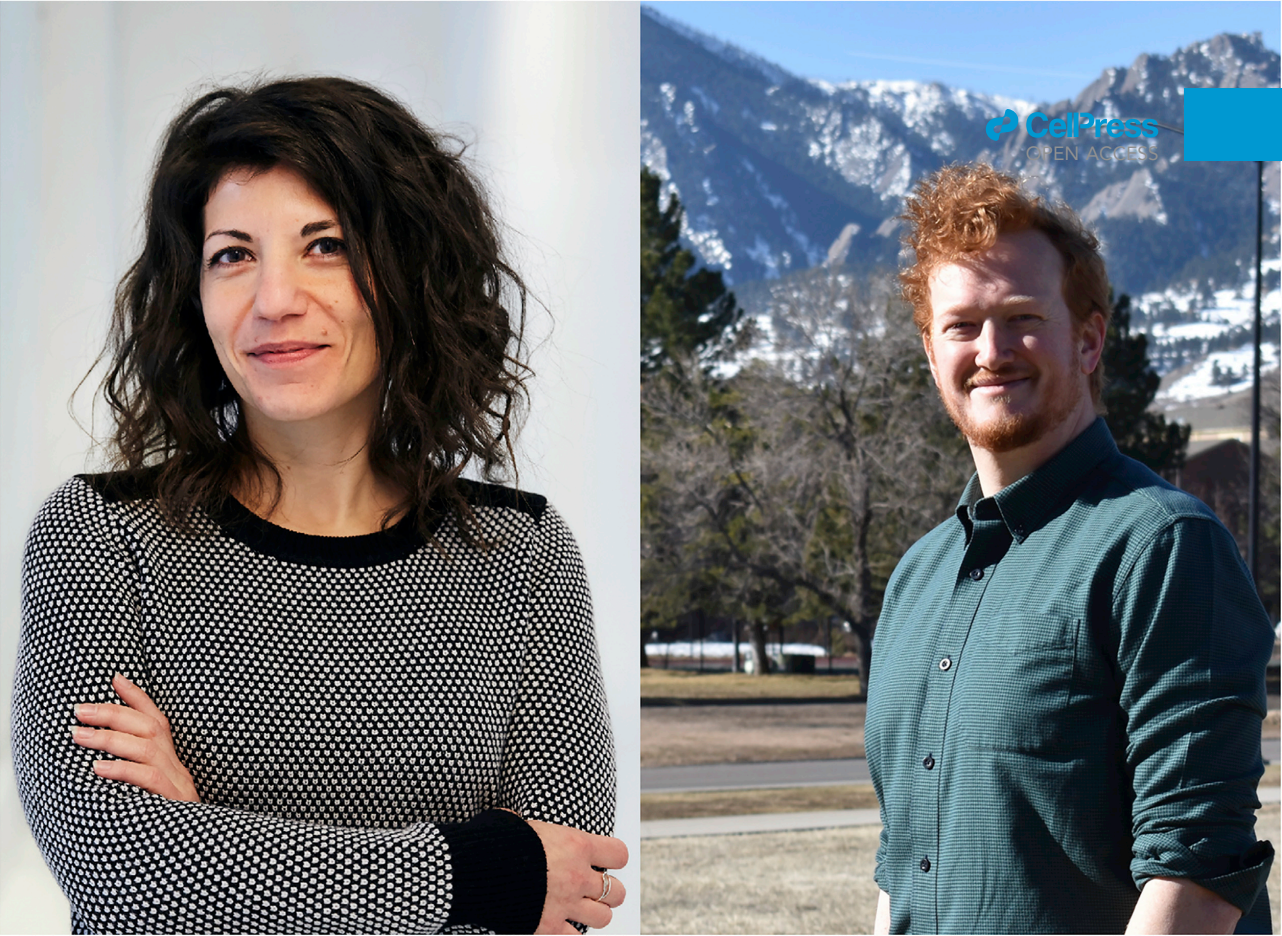
Raffaella Buonsanti (left) and Wilson Smith (right) are leaders of their fields considering the careful design of materials and engineering, respectively. In this backstory, they share their view of the field of electrochemical synthesis both as it is now, and in the future.

If you are a chemist and you are on a team with a physicist or engineer, you all look at the same problem, but you have different questions and different intuitions.

It is clear that we need collaborations within research groups but also collaborations among different groups, as there comes a time when there is a limit of what you can do internally.

When it comes to decarbonization, we have a problem we're trying to solve that needs to be done very quick and at a very large scale.

Generally, innovation comes together with a growth mindset where challenges are seen as opportunities and criticisms as a stimulus to do better. Innovation in electrosynthesis will need this growth mindset in its community at this critical stage of development.

Electricity is reshaping how we think about the future of chemistry. Due to the continued reduction in the cost of electricity generated from renewable wind and solar resources, the use of electricity to help perform chemical reactions and function in the quest toward decarbonization is becoming more attainable by the minute. However, the steps to take in using electricity to synthesize chemicals and fuels (a process known as electrochemical synthesis or electrosynthesis) are not easy. There is a need for collaboration between engineers, chemists, policy experts, and every in-between to even consider the possibilities of electrosynthesis moving from the lab to industry, where it can begin to change the energy economy.

Two scientists working on this cross talk between disciplines are Prof. Wilson Smith (NREL/CU Boulder/TU Delft) and Prof. Raffaella Buonsanti (EPFL). Combining their expertise in scale-up engineering, as well as fundamental nanoparticle catalyst design and function, Smith and Buonsanti have collaborated across labs, countries, and research disciplines to push forward the field of electrosynthesis in a tangible way.

The two have also paired up to work on a special issue with *iScience* in 2021, entitled “Emerging Pathways to Electrochemical Synthesis” which brought together articles and perspectives from many disciplinary backgrounds (https://www.sciencedirect.com/journal/iscience/special-issue/10B1SRRGD44). In the special issue, we hear from mechanical and chemical engineers, policy and grid design experts, physical, inorganic, and organic chemists, as well as industry stakeholders, all of whom bring a complementary take on this field and what is needed for electrosynthesis to emerge in the mainstream. Here we chat with Smith and Buonsanti and look to the exciting future of this field and where it is today.

## First impressions

### How do you think of the field of electrosynthesis in general as interdisciplinary?

**Smith:** When you think about the field, it is intrinsically interdisciplinary. If I think “electrosynthesis”, it can be catalysis, but then also considering the “electro-” part, it can also mean electrons, electric field, electromagnetism, and all these things we learn about in physics. Then, you have to think about the molecules coming in and out, so there is chemistry and transport, but then also there is the system as a whole, so there is engineering and then even systems process engineering.

What I like about this special issue, and in general about the field, is that the field is not a catalyst. The field is not an electrolyte. We have to understand these things, and that is the fun of this when it comes to being on a team. If you are a chemist and you are on a team with a physicist or engineer, you all look at the same problem, but you have different questions and different intuitions.

**Buonsanti:** Everyone looks at things in different ways. It is so interesting to see. Materials chemists, surface scientists, electrochemists, reactor engineers are all working together toward understanding the complex phenomena behind electrosynthesis.

Along with advanced characterization tools, theory and modeling tackling heterogeneity at different length scales are essential. Materials chemistry is essential to build tunable catalysts in a wide compositional range in order to identify the sensitivities of the performance parameters to the different features of the catalyst. The entire system includes the catalyst itself but also its support, the electrolyte, ions, gases, dissolution processes, liquids and gases that are formed, side reactions, etc. It is fascinating and stimulating to try to account for all these factors at once, but the truth is that we are still only “scratching the surface”, the more we learn the more we realize there is so much more to do.

## Proximity

### How do individual scientists or groups fit into the interdisciplinary nature of the field?

**Buonsanti:** This is something that we see in the special issue, each group focuses on a different aspect of the topic. It is clear that we need collaborations within research groups, but also collaborations among different groups, as there comes to a time when there is a limit of what you can do internally.

When I think about my research group, students and researchers come from different backgrounds; we are chemists, materials scientists, chemical engineers, and physicists. Yet, we think at the nanoscale (i.e. the catalyst and its interaction with the close surroundings). To make additional advances in the field, I believe that we must collaborate with groups that think on the device scale and the stacking of many of them. This type of desire to collaborate led to the project we worked on with Wilson. Instead of spending five years to start from scratch, a little over one year was sufficient to answer the following: can our catalysts sustain their selectivity at commercially relevant current densities? To address this research question was important to continue, and better to learn it sooner than later!

**Smith:** There are different disciplines and skills, but they have to be feeding back to each other in a self-iterating feedback loop. Process systems engineering researchers should be able to discuss about active sites and rates. Sometimes people are speaking a different language, but inherently they are talking about the same problems and the same things, so understanding this is important.

If, for example, different disciplines work on a problem in isolation, optimize their solutions, and then come together and try to fit them together, they may realize their solutions don't fit with each other in the end. Part of the “interdisciplinary” component then is that these communities need to talk to each other and inform each other at earlier stages.

## Motivation

### What motivates you and your research groups to pursue interdisciplinary science?

**Smith:** When it comes to decarbonization, we have a problem we're trying to solve that needs to be done very quick and at a very large scale. We don't have the time to accept a traditional timeline for technology development and implementation. In my group, we like to have all the information we possibly can get in this field. The interdisciplinary work, I think, helps with this timeline.

**Buonsanti**: Interdisciplinary science can be overwhelming for a first-year PhD student. They were trained in one specific discipline and when they start their research project, they need to learn about the entire picture first before diving into the details. Of course they find the big picture extremely motivating, so it eventually helps. Toward the future, thinking about how to develop a more interdisciplinary mindset at the undergrad level will become more and more important, not only for academia but also for industry.

### What are the important considerations in pursuing interdisciplinary work in electrosynthesis?

**Buonsanti:** The foundation of any collaboration requires the PIs themselves to be humble and not to pretend they know everything. Working together among groups with different expertise enables faster progress. I am a strong believer of this. There is no real need to demonstrate that you can do everything in your own group, especially when thinking about the timescale of the sustainability goals. We must move faster than we have been doing so far.

**Smith:** I think there is a definite need for more humility and open collaboration from PIs, which may be a shift from a traditional mindset. At the beginning of our collaboration with Raffaella, I was skeptical of the need for well-defined catalysts in high-current reactors, but I admired the synthetic control that Raffaella's group had in making designer catalysts. She brought all this knowledge I did not know, and then suddenly, I began to change all the ways I traditionally thought about the field. Therefore, being able to acknowledge others expertise, the value of their expertise, and then being able to adapt and change your own mindset is so important. This mindset we try to instill in our groups to work together and be a team player when it comes to these big topics, may not be the norm right now, so it is up to us to try to change to this collaborative mindset in the proposals we write and projects we pursue.

## Governance

### How do you think the governance of interdisciplinary projects (e.g. getting funding, project planning and management) impacts the research that takes place in your field?

**Smith:** I do see a difference in funding structures in Europe and the United States, especially when considering about technology readiness goals, and moving toward devices and implementation. In general, there seems to be a desire to keep multiple technologies on the table, when it is instead time to choose winners to start making substantial advances in what will actually get us to these overarching goals.

**Buonsanti:** I believe there is a lot of fundamental science to be done, which is relevant for technology. These two are essential and should feed each other. At some point industry should jump in, and with real intentions, to push the field forward. There are limits to what we can do at a university-level because our infrastructure and their scale are limited after all.

## Future

### What do you see as the future of electrosynthesis?

**Buonsanti:** As I have already mentioned, we are just getting started, the entire field is booming. The more researchers join the community the faster we will progress in different areas. Generally, innovation comes together with a growth mindset where challenges are seen as opportunities and criticisms as a stimulus to do better. Innovation in electrosynthesis will need this growth mindset in its community at this critical stage of development.

**Smith**: We must understand what the next step is. For example, we see isolated results in labs with converting pure CO_2_ to some exciting products, but we need to know this is not what will be needed in the future. Therefore, while this work can be important, understanding what the bridge is and what the next step is in the field is so important in whatever work we are doing. It is up to us as researchers and PIs to communicate to our students and lead by example that we can collaborate with others for the sake of the science. We are trying to create a disruptive technology that works better, faster, and more efficiently than what has come before it, so that requires us as a field to look at problems from all these different angles.

